# Specific Targeting of STAT3 in B Cells Suppresses Progression of B Cell Lymphoma

**DOI:** 10.3390/ijms241713666

**Published:** 2023-09-04

**Authors:** Lipei Wang, Mingqian Zhou, Xiangyu Kong, Shouzhen Wu, Chuanlin Ding, Xiaoling Hu, Haixun Guo, Jun Yan

**Affiliations:** 1 Division of Immunotherapy, The Hiram C. Polk, Jr. MD Department of Surgery, Immuno-Oncology Program, Brown Cancer Center, University of Louisville School of Medicine, Louisville, KY 40202, USA; zhoumingqian@zcmu.edu.cn (M.Z.); kxy0225@zju.edu.cn (X.K.); wushouzhen@sntcm.edu.cn (S.W.); chuanlin.ding@louisville.edu (C.D.); xiaoling.hu@louisville.edu (X.H.); 2 School of Basic Medical Sciences, Hangzhou Normal University, Hangzhou 310030, China; 3 Key Laboratory of Chinese Medicine Rheumatology of Zhejiang Province, College of Basic Medical Sciences, Zhejiang Chinese Medical University, Hangzhou 310053, China; 4 Department of Radiology, University of Louisville School of Medicine, Louisville, KY 40202, USA; haixun.guo@louisville.edu

**Keywords:** *STAT3*, SMCC, siRNA, conjugate, B cells, lymphoma

## Abstract

The signal transducer and activator of transcription 3 (*STAT3*), which regulates multiple oncogenic processes, has been found to be constitutively activated in lymphoma, suggesting its potential as a therapeutic target. Here, we constructed an anti-CD19-N-(4-carboxycyclohexylmethyl) maleimide N-hydroxysuccinimide ester (SMCC)-protamine (CSP)-*STAT3* small interfering RNA (siRNA) conjugate and demonstrated that the CSP-*STAT3* siRNA conjugate could specifically bind to normal B cells and A20 lymphoma cells in vitro. It decreased the *STAT3* expression in B cell lymphoma cell lines (A20, SU-DHL-2 and OCI-Ly3), resulting in reduced proliferation of lymphoma cells featured with lower S-phase and higher apoptosis. Using an A20 transplantable lymphoma model, we found that the CSP-*STAT3* siRNA conjugate significantly inhibited tumor growth and weight. Ki-67, p-STAT3, STAT3, and serum IL-6 levels were all significantly reduced in A20-bearing mice treated with CSP-*STAT3* siRNA. These findings indicate that specifically targeting *STAT3* siRNA to B cell lymphoma cell lines can significantly decrease *STAT3* activity and inhibit tumor progression in vitro and in vivo, suggesting its potential utilization for cancer treatment.

## 1. Introduction

STAT3 functions mainly as a transcription factor and has been shown to be involved in tumor cell proliferation, survival, and invasion in many types of human malignant tumors, including lymphoma [1,2]. Lymphoma is an aggressive lymphoid malignancy that arises primarily from lymphocytes and is heterogeneous [3]. Recent studies have shown the significant involvement of STAT3 expression in lymphoma development [4,5,6,7]. The constitutive activation of STAT3 correlates with a worse prognosis in human lymphoma [8,9]. Increased levels of IL-6, the major upstream cytokine to activate STAT3, are also associated with a poor lymphoma prognosis [10]. Collectively, these studies suggest that *STAT3* is a promising target that could be used for lymphoma therapy [11].

There are many approaches that have been developed to target *STAT3*. For example, small molecule *STAT3* inhibitors have been screened and designed. These inhibitors target STAT3 activity either via the inhibition of tyrosine kinases capable of phosphorylating and activating STAT3 or by preventing the formation of functional *STAT3* dimers through disruption of the Src Homology 2 (SH2) domains [12]. However, targeting the *STAT3* DNA binding domain and disrupting the binding of *STAT3* to its DNA promoter have been difficult. In addition, the potential off-target effects of these inhibitors may lead to unwanted adverse effects since STAT3 is a vital transcription factor that regulates many essential gene expressions related to cell cycle, cell survival, and immune response. Therefore, it is drastically needed to develop novel approaches that can specifically inhibit STAT3 activity, mainly in the target cells.

Our previous studies have shown that use of an anti-CD19 whole antibody (Ab) or mini-Ab can specifically convey antigens to B cells, leading to enhanced antitumor T and B cell responses [13,14]. In the present study, we employed this strategy to target *STAT3* siRNA into B cell lymphoma. We demonstrated that the anti-CD19-*STAT3* siRNA conjugate was able to specifically bind to B cells and inhibit STAT3 activity. With the use of an A20 lymphoma model, we showed that this conjugate could specifically inhibit the overexpressed STAT3 activity in lymphoma cells. Further in vivo studies showed that anti-CD19-*STAT3* siRNA conjugate significantly reduced tumor progression with decreased STAT3 activity, suggesting potential utilization for cancer treatment.

## 2. Results

### 2.1. Development of CD19 mAb-SMCC-Protamine-siRNA Conjugate System

**Figure 1:**
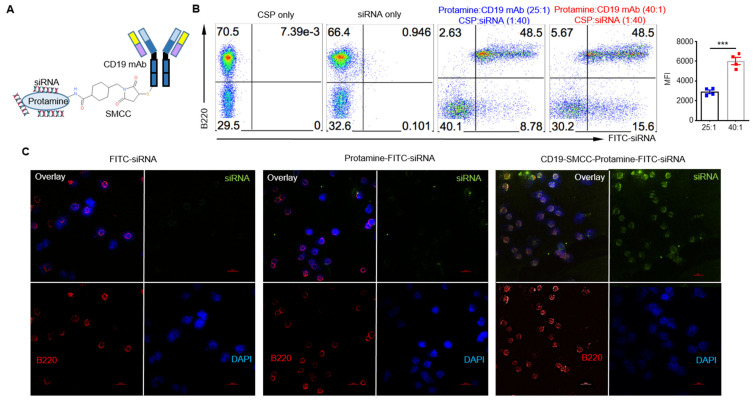
Properties of the CSP-specific siRNA carrier system: (**A**) The graphical depiction of anti-CD19 mAb coupling to protamine by bivalent cross-linker sulfo-SMCC siRNA conjugate via electrostatic interactions. (**B**) Flow cytometric analysis showed the affinity of the CSP-FITC-labeled siRNA conjugate to B cells. Representative dot plots and summarized MFI data are shown. MFI: mean fluorescent intensity. Pseudocolor plots are a type of bivariate density plot. It displays the relative population density of cell populations within the Graph Window along two parameters. Data are shown as the mean ± SEM (*n* = 3). *** *p* < 0.001, Student’s *t*-test. Red dots are from protamine:CD19 mAb at 40:1 ratio while blue dots are at 25:1 ratio. (**C**) Confocal laser scanning microscopy images of the FITC-siRNA (green), protamine FITC-siRNA, and CSP FITC-siRNA conjugate staining in B cells (B220, red). DAPI staining (blue) was used to reveal nuclei. Scale bars: 20 μm.

The cell-type-specific delivery of siRNA is a major challenge for siRNA-based therapies [15,16]. Previous studies have tested several methods to couple antibodies with siRNA, such as use of protamine as an siRNA-complexing agent [17]. Protamine is a positively charged molecule, while siRNA carries a negative charge, leading to electrostatic interactions. CD19 is a molecule specifically expressed by B lymphocytes. Our previous studies have shown that targeting antigens (Ags) to B cells via CD19 mAb or mini-Ab can induce augmented T cell and humoral responses [13,14]. To this end, we first coupled protamine to CD19 mAb using a sulfo-SMCC linker in a 5:1 molar ratio, enabling the binding of multiple protamine molecules per molecule of mAb, and then conjugated this with siRNA (Figure 1A). The CD19 mAb-SMCC-protamine conjugates were designated as a CSP. To examine whether the CSP-siRNA conjugates specifically bind to B cells, we used splenocytes and then mixed them with different molar ratios of CSP-Fluorescein Isothiocyanate (FITC)-labeled siRNA conjugate. As shown in Figure 1B, CSP-siRNA conjugates were specifically bound to B cells, and increasing the ratio of protamine did not significantly increase the percentage of B cell binding, but mean fluorescent intensity (MFI) was increased, suggesting that more siRNAs were bound to the CSP conjugates. The binding was specific since siRNA alone did not show any binding to B cells. A confocal microscopic analysis was performed to further evaluate the efficiency of conjugate binding to purified B cells in vitro. Indeed, FITC-labeled siRNA-CSP conjugates showed specific binding to B cells, while protamine-siRNA conjugate or siRNA alone did not show any binding to B cells (Figure 1C). Taken together, these data suggest that we are able to specifically deliver siRNA to B cells through the generation of CD19 mAb-protamine-siRNA conjugates.

### 2.2. STAT3 Expression on B Cell Lymphoma Cells Was Decreased by CSP-STAT3 siRNA Conjugate

**Figure 2:**
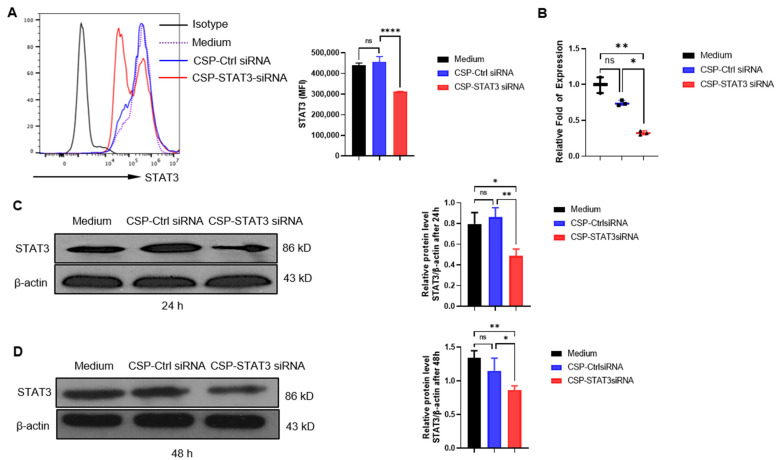
CSP-*STAT3* siRNA conjugate suppresses STAT3 expression in A20 cells: (**A**) A20 cells (1 × 10^6^) were incubated with RPMI 1640 medium (Medium), 60 nM CSP-control siRNA conjugate (CSP-Ctrl siRNA), or 60 nM CSP-*STAT3* siRNA conjugate (CSP-*STAT3* siRNA) for 24 h at 37 °C. Flow cytometric analysis was performed for STAT3 expression (MFI, mean fluorescent intensity; *n* = 3 for each group). Shown here is one representative of three independent experiments. **** *p* < 0.0001, one-way ANOVA. (**B**) A20 cells treated as above were collected and total RNA was isolated for qRT-PCR. Data are shown as the mean ± SEM (*n* = 3). * *p* < 0.05; ** *p* < 0.01; one-way ANOVA. (**C**,**D**) After 24 h (**C**) and 48 h (**D**) treatment, A20 cells were lysed and proteins were extracted for Western blot and quantification analysis of relative STAT3 (*n* = 3/group). Shown here is one representative of three independent experiments. ns, not significant; * *p* < 0.05; ** *p* < 0.01; one-way ANOVA.

STAT3 is overexpressed in lymphoma cells and the expression level correlates with a worse prognosis [18,19]. As the CSP-specific siRNA conjugate was effectively taken up by B cells, we next examined the intracellular effect of *STAT3* RNA interference (RNAi) by the CSP-*STAT3* siRNA conjugate. The best *STAT3* RNAi was selected based on its knockdown efficiency for further studies (Supplementary Figure S1). Three B cell lymphoma cell lines were incubated with 60 nM CSP-*STAT3* siRNA conjugate or 60 nM CSP-control siRNA conjugate for 24 h or 48 h. As shown in Figure 2A, the A20 cells incubated with CSP-*STAT3* siRNA conjugates but not CSP-control siRNA exhibited significantly reduced STAT3 expression levels, as assessed by flow cytometry. In addition, the mRNA expression levels (Figure 2B) and protein expression levels of STAT3 (Figure 2C) were also significantly reduced by the CSP-*STAT3* siRNA conjugates. The inhibitory effect of the CSP-*STAT3* siRNA conjugates lasted even after 48 h of treatment (Figure 2D). The SU-DHL-2 and OCI-Ly3 lymphoma cells incubated with CSP-*STAT3* siRNA conjugates also exhibited significantly reduced STAT3 expression (Supplementary Figure S2). These results suggest that CSP-*STAT3* siRNA conjugates can inhibit STAT3 expression effectively.

### 2.3. CSP-STAT3 siRNA Conjugates Inhibit B Cell Lymphoma Cell Cycle and Induce Apoptosis

**Figure 3:**
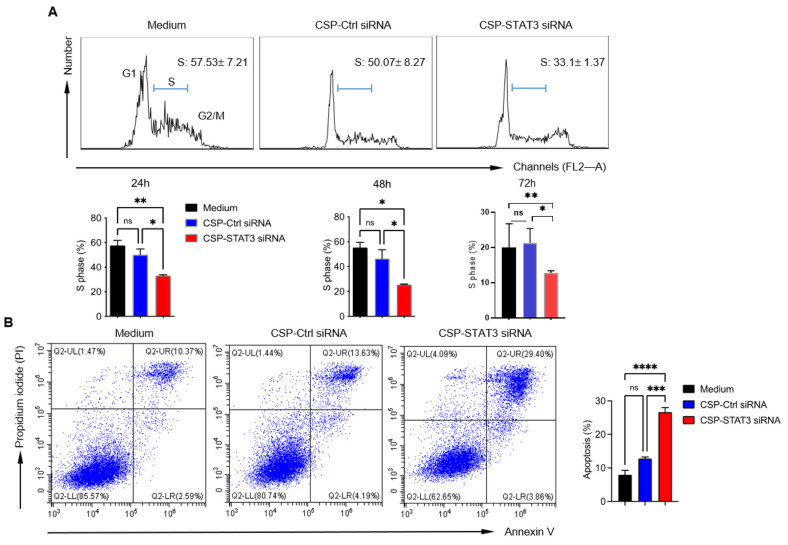
CSP-*STAT3* siRNA conjugate inhibits A20 lymphoma cell cycle and induces apoptosis of A20 cells: (**A**) A20 cells (1 × 10^6^) were incubated with RPMI 1640 medium (Medium), 60 nM CSP-control siRNA conjugate (CSP-Ctrl siRNA), or 60 nM CSP-*STAT3* siRNA conjugate (CSP-*STAT3* siRNA) for 24 h at 37 °C. Flow cytometric analysis was performed for cell cycle analysis. Representative flow plots and summarized data are shown as the mean ± SEM (*n* = 3). (**B**) A20 cells (1 × 10^6^) were incubated with RPMI 1640 medium, 60 nM CSP-control siRNA conjugate, or 60 nM CSP-*STAT3* siRNA conjugate for 24 h at 37 °C. Apoptosis assay was performed and analyzed using flow cytometry. Representative flow plots and summarized data are shown (*n* = 3). ns, not significant; * *p* < 0.05; ** *p* < 0.01; *** *p* < 0.001; **** *p* < 0.0001, one-way ANOVA.

STAT3 is a critical transcription factor and regulates many genes related to cell cycle and proliferation [20]. We thus analyzed the effects of different conjugates on the cell cycle of A20, SU-DHL-2, and OCI-Ly3 lymphoma cells. The mammalian cell cycle consists of four distinct phases: the G1 phase, the S (synthesis) phase, and the G2/M phase. DNA replication occurs during the S phase. As shown in Figure 3A and Supplementary Figure S3, the CSP-*STAT3* siRNA conjugates, but not the CSP control siRNA conjugates, significantly reduced the S phase. This effect lasted even 72 h after the treatment of the A20 cells. The CSP-*STAT3* siRNA conjugates also exhibited a reduction effect in the S phase in SU-DHL-2 and OCI-Ly3 cells after 24 h of treatment (Supplementary Figure S4). To examine whether the inhibition of *STAT3* induces lymphoma cell apoptosis, we treated the A20, SU-DHL-2, and OCI-Ly3 cells with different conjugates. All three cell lines treated with CSP-*STAT3* siRNA conjugates underwent more apoptosis (Figure 3B and Figure S5). These data indicate that the specific targeting of *STAT3* siRNA to B cell lymphoma can reduce lymphoma cell proliferation and induce apoptosis in vitro.

### 2.4. Treatment of CSP-STAT3 siRNA Conjugates Reduces A20 Lymphoma Progression in NOD SCID Mouse Transplanted Tumor

**Figure 4:**
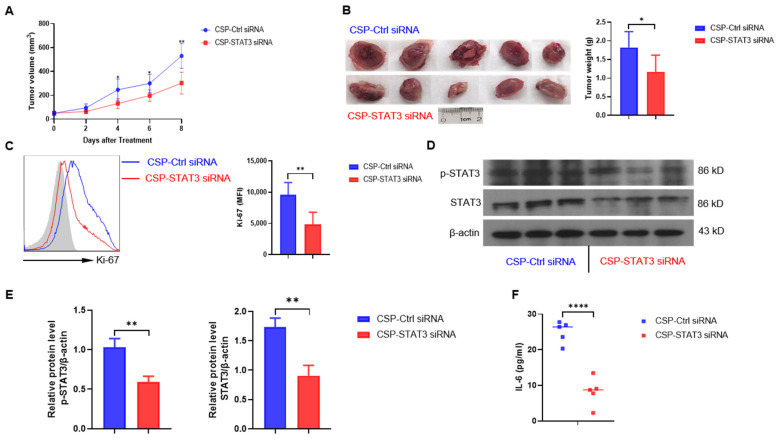
Anti-tumor activity of CSP-*STAT3* siRNA conjugate in NSG mice: (**A**) A20 cells (2.0 × 10^6^) were subcutaneously implanted in NSG mice. After tumors reached a palpable size, mice (*n* = 5) were treated with CSP-*STAT3* siRNA conjugate (CSP-*STAT3* siRNA) or CSP-control siRNA conjugate (CSP-Ctrl siRNA) in vivo every two days. Tumor growth was followed with caliper measurements in a blinded fashion. * *p* < 0.05; ** *p* < 0.01, Student’s *t*-test. (**B**) At the end of the experiment, animals were euthanized, primary tumors were surgically removed, and tumor weight was determined (*n* = 5). * *p* < 0.05, Student’s *t*-test. (**C**) The expression of the proliferation antigen Ki-67 was markedly reduced in A20 transplanted tumors from NSG mice treated with CSP-*STAT3* siRNA conjugate, using flow cytometric analysis (MFI, mean fluorescent intensity; *n* = 5 for each group). ** *p* < 0.01, Student’s *t*-test. (**D**) Western blot analysis was performed for STAT3, p-STAT3, and actin as loading control. Three tumor samples from each group are shown. (**E**) Quantitative analysis of the relative expression level of proteins as shown in (**D**). ** *p* < 0.01, Student’s *t*-test. (**F**) Serum IL-6 levels from A20-bearing mice treated with control or CSP-*STAT3* siRNA were determined by ELISA (*n* = 5). **** *p* < 0.0001, Student’s *t*-test.

Next, we analyzed the effect of CSP-*STAT3* siRNA conjugate in vivo. To test the therapeutic effect of the CSP-*STAT3* siRNA conjugate treatment, A20 cells were subcutaneously injected into NOD SCID (NSG) mice, and mice with palpable tumors were randomized into two groups. Tumor-bearing mice were treated every two days by an in vivo injection of either CSP-*STAT3* siRNA conjugate or CSP-control siRNA conjugate, and tumor growth was monitored. The CSP-*STAT3* siRNA conjugate significantly inhibited tumor growth compared with the CSP-control siRNA conjugate (Figure 4A). This was also revealed by analyzing tumor weight (Figure 4B). Further experiments were performed to analyze Ki-67 as a proliferation marker in the tumors from mice treated in vivo. In the CSP-*STAT3* siRNA conjugate tumors, Ki-67 was significantly reduced as compared to those from the CSP-control siRNA-conjugate-treated mice (Figure 4C). We also detected STAT3 expression by carrying out a Western blot analysis on the tumors after CSP-*STAT3* siRNA conjugate or CSP-control siRNA conjugate treatment. In the A20 tumors treated with CSP-*STAT3* siRNA conjugate, both the p-STAT3 and STAT3 levels were suppressed compared to those treated with CSP-control siRNA conjugates (Figure 4D,E). In addition, we found that the serum IL-6 level was also significantly reduced in mice treated with CSP-*STAT3* siRNA conjugates (Figure 4F). Taken together, these findings suggest that specifically targeting *STAT3* siRNA to B cell lymphoma cells can significantly reduce tumor burden and progression.

## 3. Discussion

In this study, we developed a conjugate comprising *STAT3* siRNA and CD19 mAb, which selectively bind to B cells. We showed that this conjugate specifically targets B cells and inhibits *STAT3* signaling activation. Both in vitro and in vivo studies revealed that this approach can effectively inhibit A20 B cell lymphoma proliferation and tumor progression. In addition, targeting *STAT3* with this strategy also leads to decreased cell proliferation and increased apoptosis in two other B cell lymphoma cell lines.

Persistent *STAT3* signaling activation promotes tumor cell proliferation, survival, and metastasis. In addition, *STAT3* activation leads to tumor-promoting inflammation and suppresses antitumor immunity [2,21]. Previous studies demonstrated that direct inhibition of *STAT3*, rather than indirect inhibition via targeting upstream signaling, may be more effective as a therapeutic strategy for tumors by using the shRNA approach [22]. However, the delivery of shRNA requires the use of a lentivirus vector, which complicates future clinical translation. In addition, a TLR9 agonist fused to a *STAT3* decoy oligodeoxynucleotide (dODN) was also used to inhibit *STAT3* in B cell lymphoma [23]. Furthermore, many efforts have been made to develop approaches to effectively inhibit *STAT3* signaling activation in the tumor microenvironment. However, the specific targeting of *STAT3* has been challenging. This is particularly critical since *STAT3* signaling is also essential in hair follicle and bone homeostasis [24,25]. siRNAs, used for the specific down-regulation of targeted genes, have garnered considerable interest as an attractive new class of drugs for a broad range of clinical applications [26]. The polyanionic charges carried by these siRNAs, however, restrain their cellular uptake and consequently limit their effects on gene regulation. In the current study, anti-CD19 was covalently conjugated to *STAT3* siRNA using SMCC and protamine. The binding of the CD19-SMCC-protamine-*STAT3* siRNA conjugate in vitro was tested using splenic lymphocytes and the lymphoma A20 cell line originated from B cells. Analyses of in vitro data showed that the CD19-SMCC-protamine-*STAT3* siRNA conjugate silenced STAT3 expression effectively, leading to decreased A20 in vitro proliferation and increased apoptosis. It appears that CSP conjugate alone also had a minimal effect on the lymphoma cell cycle and apoptosis induction, although it is not statistically different from the untreated group. Our previous studies have shown that antigens directly targeted to the CD19 protein on B cells via anti-CD19 alone can be internalized and form a cap at the B cell surface through CD19 [27]. In addition, other studies also confirmed the clinical implications of CD19 as an immunotherapeutic target for cancer [28]. Thus, it is possible that CSP alone may have some levels of effect directly via the CD19 molecule. However, CSP-*STAT3* siRNA conjugates result in a more potent effect on A20 cell proliferation and apoptosis induction. The administration of the CD19-SMCC-protamine-*STAT3* siRNA conjugate in vivo also led to the inhibition of A20 lymphoma progression. It is worth noting that this conjugate did not completely abolish STAT3 protein expression. This may be related to the siRNA knockdown efficiency.

Previous studies indicate that an IL-6-STAT3 positive feedback loop was involved in tumorigenesis [29]. Indeed, STAT3 was further activated by IL-6 after being treated with 15 min in A20 lymphoma. Interestingly, the knockdown of *STAT3* in A20 lymphoma also leads to a reduced IL-6 production in tumor-bearing mice. We speculate that a low level of IL-6 further diminishes STAT3 activation, thereby reducing tumor progression. It is worth noting that this conjugate was used in the setting of immunocompromised mice, which do not have normal B cells. In the future, we need to test this approach in an immunocompetent mouse tumor model to examine whether this approach also impacts normal B cell function. Alternatively, it remains to be explored whether the efficacy is diminished due to the conjugate binding to normal B cells. In addition, a comprehensive toxicology study should be performed on mice, although previous studies have shown that both SMCC and protamine are proven to be safe for in vivo use [30,31], and we did not detect the toxic effect of conjugate in our study.

## 4. Materials and Methods

### 4.1. Coupling of Anti-CD19 mAb to Protamine Sulfate by SMCC

Protamine sulfate (2 mg/mL in PBS) was amino-terminally coupled to the bifunctional cross-linker Sulfo-SMCC (2 mg/mL in PBS) at 1:5 molar ratio for 1 h at room temperature on a shaker. PD Spin Trap G25 column (GE Healthcare No.28-9180-04, Chicago, IL, USA) was used to remove uncoupled protamine or SMCC. Conjugates were then mixed with anti-mouse CD19 mAb (32 umol/L, BioXcell, Lebanon, NH, USA) in a 25:1 molar ratio at 4 °C overnight. Non-reacted products and protamine doublets were separated from anti-CD19-SMCC-protamine product using NAP-10 desalting columns (GE Healthcare No.17-0854-01). The anti-CD19-SMCC-protamine conjugates were stored at 4 °C and were stable for several months.

### 4.2. siRNA

*STAT3* siRNA (sense strand: 5′-GUUGAAUUAUCAGCUUAAA-3′; anti-sense strand: 5′-UUUAAGCUGAUAAUUCAAC-3′). Negative control siRNA (SIC001, mission siRNAs) and FITC-conjugated control siRNA were purchased from Sigma-Aldrich (St. Louis, MO, USA).

### 4.3. Coupling of siRNA to Anti-CD19-SMCC-Protamine Conjugates

The siRNAs were bound to anti-CD19-SMCC-protamine conjugates in a 1:10 molar ratio at room temperature for 1–2 h. This complex was prepared freshly before use.

### 4.4. Flow Cytometric Analysis

Splenocytes treated with CSP, siRNA, and different conjugates for 30 min were stained with B220-APC and FITC-conjugated siRNA to examine B cell binding. A20 cells with different treatments for 24 h were stained with anti-STAT3-PE (BioLegend, San Diego, CA, USA). Cells were acquired on a FACSCanto flow cytometer (BD Biosciences, Franklin Lakes, NJ, USA) and analyzed with analyzed using FlowJo software v.1 (Tree Star, Ashland, OR, USA). FACSDiva software version 6 was used in FACSCanto to acquire all data.

### 4.5. Confocal Microscopy

Purified B cells were plated on Lab-Tek (4-well) chambered cover glasses at a density of 1 × 10^4^ cells/cover glass and incubated at 37 °C in humidified 5% CO_2_. FITC-siRNA, protamine FITC-siRNA, and anti-CD19-SMCC-protamine-FITC-siRNA conjugate were overlaid onto B cells for 30 min and then further incubated with 4′,6-diamidino-2-phenylindole (DAPI) to reveal nuclei. Slides were observed under the Leica Confocal Microscopy (Leica Microsystems, Wetzlar, Germany).

### 4.6. Cell Culture

Mouse lymphoma A20 cells were purchased from ATCC. SU-DHL-2 and OCI-Ly3 cells were provided by Professor Zhengrong Mao of Zhejiang University. All cells were cultured in RPMI 1640 medium supplemented with 100 units/mL penicillin G and 0.1 mg/mL streptomycin containing 10% fetal bovine serum (FBS) under a 5% CO_2_ atmosphere at 37 °C.

### 4.7. RNA Purification and qRT-PCR

Total RNAs from A20 cells with different treatments for 24 h were extracted using Trizol. RNA was used for reverse-transcription with an iScript™ cDNA Synthesis Kit (Bio-Rad, Hercules, CA, USA) according to the manufacturer’s instructions. For qRT-PCR, the reaction mixtures (20 µL) contained 10 µL of iQ™ SYBR^®^ Green Supermix (Bio-Rad), 0.5 µM of forward and reverse primers, and 0.2 µL of cDNA. PCR conditions were 95 °C for 5 min (one cycle), 95 °C for 10 s, 60 °C for 30 s (39 cycles), 65 °C for 7 s and a ramp to 95 °C (one cycle). STAT3 expression level was normalized to *β-MG* for *STAT3* siRNA sample and calculated relative to control siRNA sample using the following equation.

Fold change = 2^−(STAT3ΔCt_controlΔCt)^, where ΔCt = Ct^(STAT3/control)^ − Ct^(BMG)^.

Primer sequences for qRT-PCR analysis:

STAT3 (forward: 5′-GTCTGCAGAGTTCAAGCACCT-3′; reverse: 5′-TCCTCAGTCACGATCAAGGAG-3′)

BMG (forward: 5′-CTTTCTGGTGCTTGTCTC-3′; reverse: 5′-TCAGTATGTTCGGCTTCC-3′)

### 4.8. Western Blot Analysis

Cells were lysed in ice-cold Lysis Buffer (Cell Signal Technology, Danvers, MA, USA) with Phosphatase Inhibitor Cocktail Set III (Sigma-Aldrich). Total protein was analyzed for the protein concentration using a bicinchoninic acid (BCA) kit (Bio-Rad, Hercules, CA, USA). The protein samples were added to loading buffer, boiled for 5 min, and loaded onto gels at 60 μg/well. Next, the proteins were isolated with 10% sodium dodecyl sulfate-polyacrylamide gel electrophoresis (SDS-PAGE), transferred onto polyvinylidene fluoride (PVDF) membranes, and blocked with 5% BSA at room temperature for 1 h. PVDF membranes were incubated overnight at 4 °C with the following primary antibodies: anti-phospho STAT3 (Sc-482, Santa Cruz biotechnology, Dallas, TX, USA), anti-STAT3 (9145S, Cell Signaling Technology, Danvers, MA, USA), and β-Actin antibody (Sigma-Aldrich). The next day, the membranes were washed with TBST 3 times/5 min, followed by the addition of the corresponding secondary antibody for a 1 h incubation. The membranes were washed again with TBST 3 times/5 min before the chemiluminescence (CL) reaction. Blots were analyzed using Image J software version 1.8.0.

### 4.9. Cell Cycle Measurement

Cells (0.5 × 10^6^) were first treated with different conjugates or controls at indicated times and were washed with PBS buffer and fixed in 1 mL of ice-cold 70% ethanol overnight at 4 °C. The next day, cells were centrifuged at 2000 rpm and pellets were washed with PBS buffer. After removing the supernatant, RNase A (100 U/mL final) was added in samples and incubated at 37 °C for 1–3 h. Next, 0.25 mL of PBS buffer and 1 mg/mL of Propidium Iodide (PI) were added, followed by incubation at room temperature for 30 min, avoiding exposure to light. The cell cycle was analyzed using a FACSCalibur^TM^ Flow Cytometer (BD Biosciences, Franklin Lakes, NJ, USA).

### 4.10. Apoptosis Assay

Cells treated with different conjugates or controls for 24 h were collected. Cells were washed twice with cold BioLegend’s Cell Staining Buffer and then resuspended in Annexin V Binding Buffer at a concentration of 0.25–1.0 × 10^7^ cells/mL. Next, 5 µL of APC Annexin V and 10 µL of Propidium Iodide Solution were added. Gently, the cells were vortexed and incubated for 15 min at room temperature (25 °C) in the dark. Then, 400 µL of Annexin V Binding Buffer was added to each tube. The cell apoptosis was detected with a FACSCalibur^TM^ Flow Cytometer.

### 4.11. Tumor Model Establishment

For tumor study, NSG mice (6–8 weeks old) were inoculated subcutaneously with 100 µL of a single-cell suspension containing A20 cells (2 × 10^6^/mouse). When tumor size reached about 50–100 mm^3^ (approximately 10 days after tumor implantation), mice were randomized into different groups for treatments. The mice bearing A20 tumors were injected intravenously with CSP-control siRNA conjugates or CSP-*STAT3* siRNA conjugates (120 nM/per mouse) for 5 times every other day. Tumor diameters were measured with a caliper and tumor volumes were calculated using the following formula: volume = 0.52 × length × (width)^2^, where length represented the longest tumor diameter and width represented the shortest tumor diameter. Mice were euthanized 2 days after the last treatment and the tumors were excised and weighed. All experimental mice were housed under specific pathogen-free conditions in the animal facility of the University of Louisville and treated in accordance with the guidelines of the Institutional Animal Care and Use Committee (IACUC) of the University of Louisville.

### 4.12. Ki-67 Staining

For Ki-67 staining, single-cell suspensions from tumors were incubated with Fix/Perm solution for 30 min in the dark at room temperature. Cells were then washed and Perm buffer was added. After being washed three times, cells were incubated with PE-labeled anti-Ki-67 antibodies for 1 h. The Ki-67 expression was assessed with flow cytometry.

### 4.13. IL-6 ELISA

The serum was collected from A20-bearing mice, and IL-6 level was measured using an IL-6 ELISA kit (BioLegend) according to the manufacturer’s instructions.

### 4.14. Statistical Analysis

All data are presented as mean ± SEM, if not indicated otherwise. The mean values of two groups were compared using the Student’s *t* test. For both groups, ordinary one-way ANOVA (parametric) was used for the statistical analysis. *p* < 0.05 was considered to be statistically significant.

## Data Availability

The data underlying this article will be shared upon reasonable request to the corresponding author.

## Supplementary Materials

The following supporting information can be downloaded at: https://www.mdpi.com/1422-0067/24/17/13666/s1.
