# Supplementary material for: Specific Targeting of STAT3 in B Cells Suppresses Progression of B Cell Lymphoma
Source: Int J Mol Sci. 2023 Sep 4;24(17):13666. doi: 10.3390/ijms241713666 (PMC10563066; doi:10.3390/ijms241713666)
Supplement: Supplementary file 1 [file ijms-24-13666-s001.zip › ijms-2390960-supplementary.pdf]

International Journal of *Molecular Sciences*

MDPI

Supplementary Figure S1

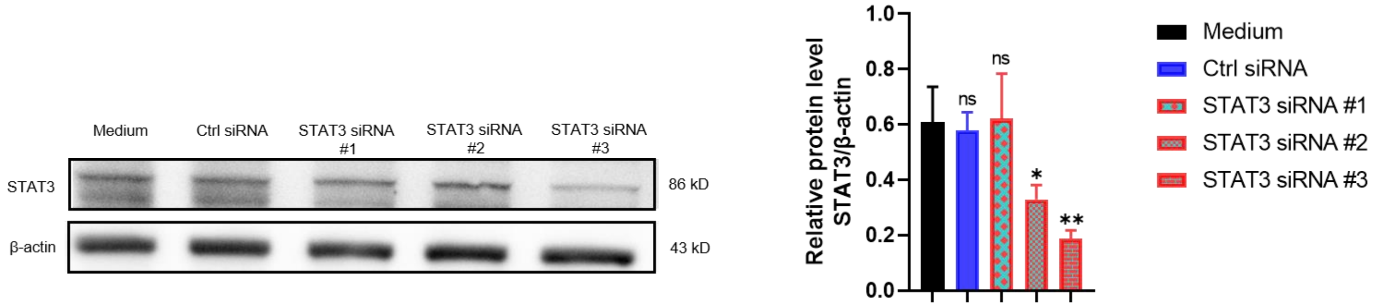

Supplementary Figure S1. STAT3 expression of A20 lymphoma cell treated by *STAT3* siRNA. A20 cells (1×10^6) were incubated with RPMI 1640 medium (Medium), Control siRNA (Ctrl siRNA) or three different *STAT3* siRNAs (*STAT3* siRNA #1, #2, #3) for 24 h at 37℃. A20 cells were lysed and proteins were extracted for Western blot and quantification analysis of relative STAT3 ($n = 3$/group). One representative WB analysis and summarized data from three independent experiments are shown. ns, not significant; $^{*}P<0.05$; $^{**}P<0.01$, compared with Medium group; Student's *t*-test.

Supplementary Figure S2

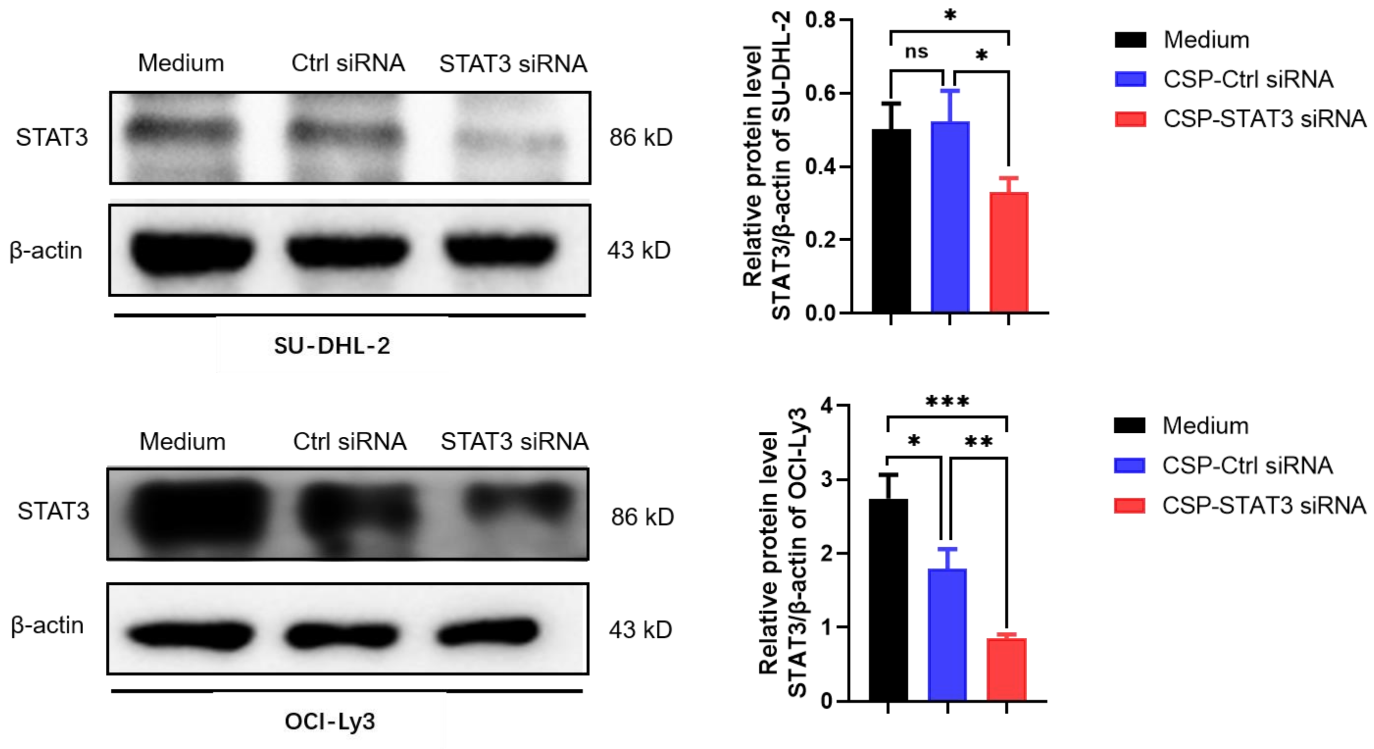

Supplementary Figure S2. CSP-*STAT3* siRNA conjugate inhibits STAT3 expression in SU-DHL-2 and OCI-Ly3 cells. SU-DHL-2 and OCI-Ly3 cells (1×10^6 cells of each cell line) were incubated with RPMI 1640 medium (Medium), 60 nM CSP-Control siRNA conjugate (CSP-Ctrl siRNA) or 60 nM CSP-*STAT3* siRNA conjugate (CSP-*STAT3* siRNA) for 24 h

at 37℃. Cells were lysed and proteins were extracted for Western blot and quantification analysis of relative STAT3 (*n* = 3/group). Shown here is one representative of three independent experiments. ns, not significant; **P*<0.05; ***P*<0.01; ****P*<0.001, one-way ANOVA.

Supplementary Figure S3

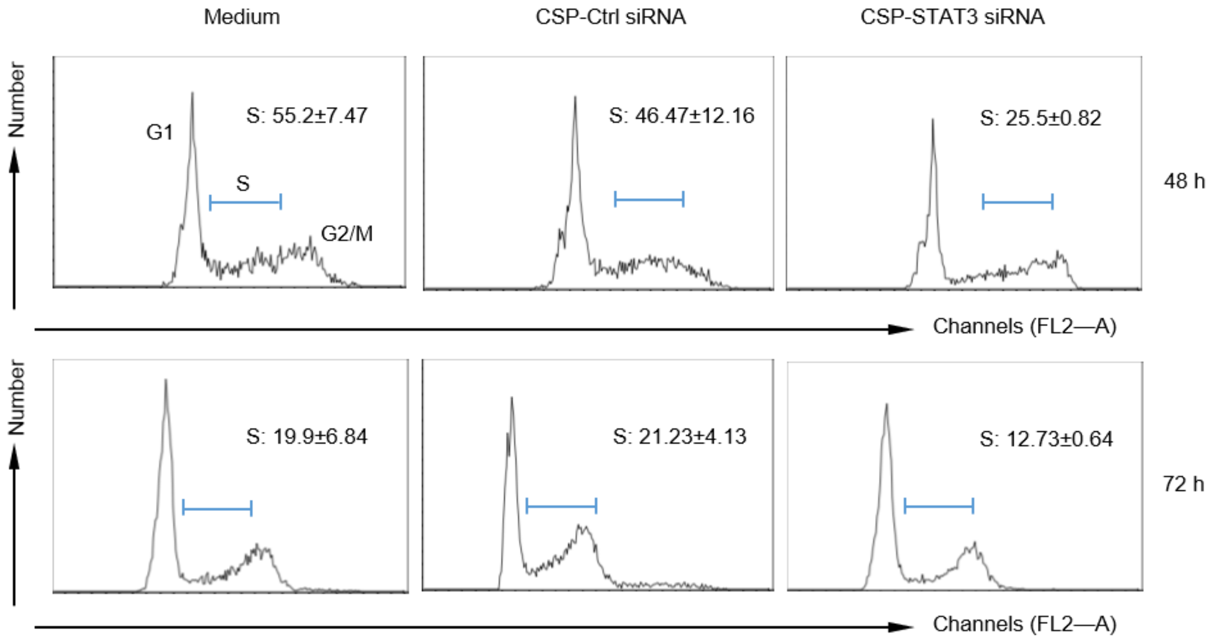

Supplementary Figure S3. CSP-*STAT3* siRNA conjugate inhibits A20 lymphoma cell cycle. A20 cells (1×10^6) were incubated with RPMI 1640 medium (Medium), 60 nM CSP-Control siRNA conjugate (CSP-Ctrl siRNA) or 60 nM CSP-*STAT3* siRNA conjugate (CSP-*STAT3* siRNA) for 48 h or 72 h at 37°C. Flow cytometric analysis was performed for cell cycle analysis. Representative flow plots are shown.

Supplementary Figure S4

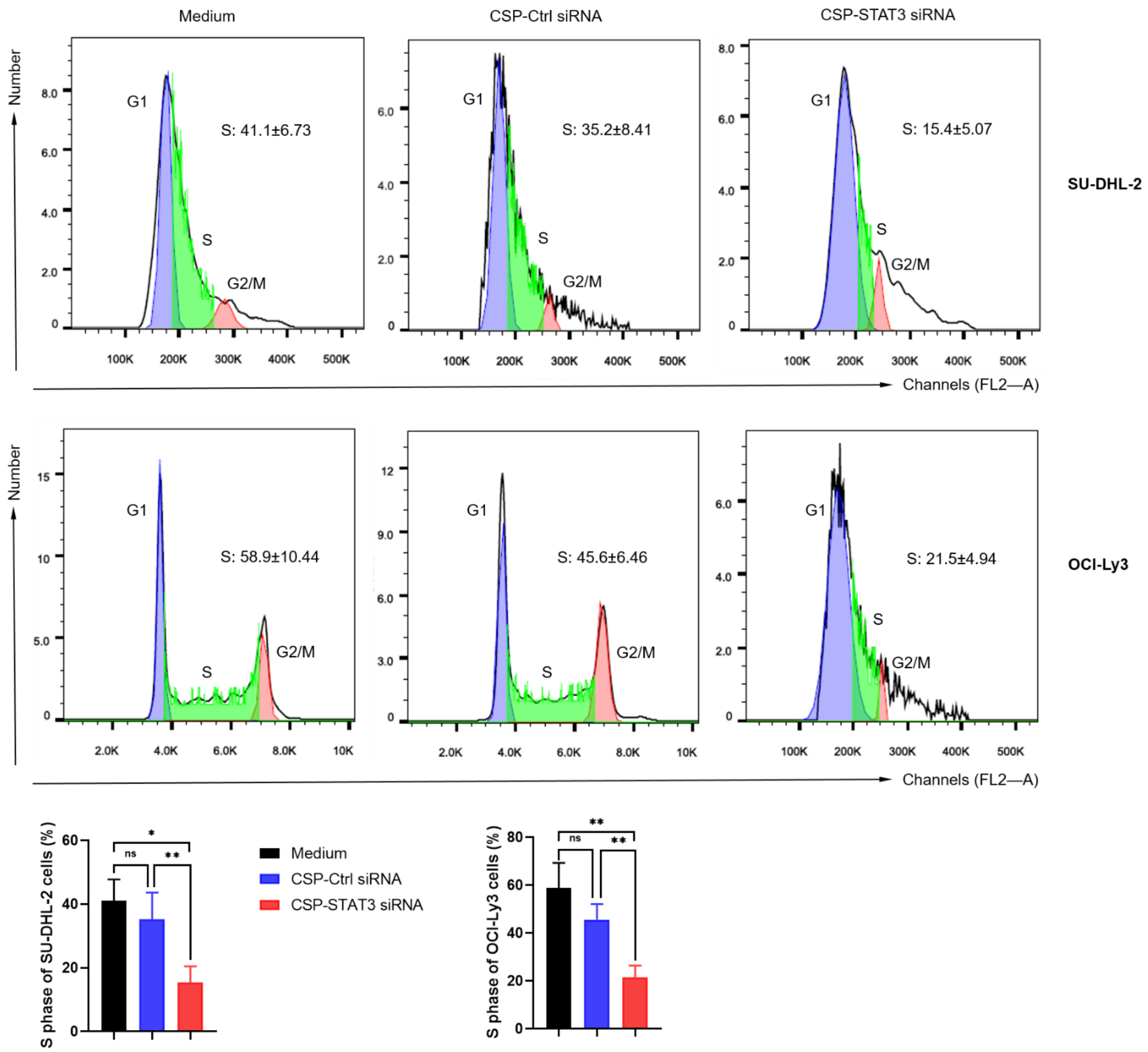

Supplementary Figure S4. CSP-*STAT3* siRNA conjugate inhibits cell cycle of SU-DHL-2 and OCI-Ly3 cells. SU-DHL-2 and OCI-Ly3 cells (1×10^6) were incubated with RPMI 1640 medium (Medium), 60 nM CSP-Control siRNA conjugate (CSP-Ctrl siRNA) or 60 nM CSP-*STAT3* siRNA conjugate (CSP-*STAT3* siRNA) for 24 h at 37℃. Flow cytometric analysis was performed for cell cycle analysis. Representative flow plots and summarized data are shown as the mean ± sem (n = 3). ns, not significant; *P<0.05; **P<0.01, one-way ANOVA.

Supplementary Figure S5

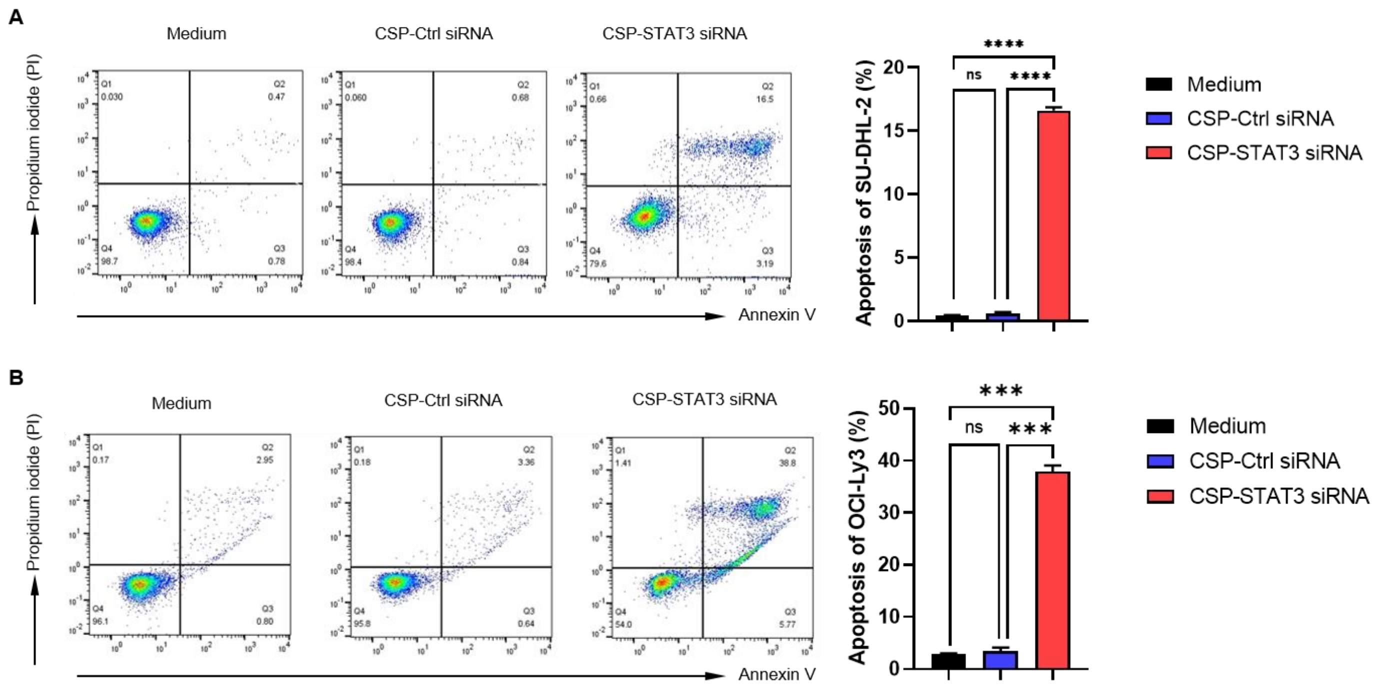

Supplementary Figure S5. CSP-*STAT3* siRNA conjugate induces apoptosis of SU-DHL-2 and OCI-Ly3 cells. **(A)** SU-DHL-2 cells (1×10^6) were incubated with RPMI 1640 medium (Medium), 60 nM CSP-Control siRNA conjugate (CSP-Ctrl siRNA) or 60 nM CSP-*STAT3* siRNA conjugate (CSP-*STAT3* siRNA) for 24 h at 37℃. Apoptosis assay was performed and analyzed by flow cytometry. Representative flow plots and summarized data are shown (n = 3). ns, not significant; $^{****}P<0.0001$, Student's t-test. **(B)** OCI-Ly3 cells (1×10^6/group) were treated with or without CSP-Ctrl siRNA and CSP-*STAT3* siRNA for 24 h at 37℃. Apoptosis assay was performed and analyzed by flow cytometry. Representative flow plots and summarized data are shown (n = 3). ns, not significant; $^{***}P<0.001$, Student's t-test.
